# Serological Profile of Children and Young Adults with at Least One SARS-CoV-2 Positive Cohabitant: An Observational Study

**DOI:** 10.3390/ijerph18041488

**Published:** 2021-02-04

**Authors:** Marco Farronato, Carolina Dolci, Elisa Boccalari, Sara Izadi, Luis Hernan Salvatierra Rios, Maurizio Festa, Valentina Panetta, Danila De Vito, Gianluca Martino Tartaglia

**Affiliations:** 1 Department of Biomedical, Surgical and Dental Sciences, School of Dentistry, University of Milan, 20122 Milan, Italy; marco.farronato@unimi.it (M.F.); carolina.dolci@unimi.it (C.D.); eli.boccalari@gmail.com (E.B.); sara.izadi@unimi.it (S.I.); luis.salvatierra@unimi.it (L.H.S.R.); maurizio.festa@unimi.it (M.F.); 2 Fondazione IRCCS Cà Granda, Ospedale Maggiore Policlinico, 20122 Milan, Italy; 3 L’Altrastatistica Consultancy & Training, Biostatistics Office, 00175 Rome, Italy; valentina.panetta@laltrastatistica.com; 4 Department of Basic Medical Sciences, Neurosciences and Sense Organs, University of Bari “Aldo Moro”, 70124 Bari, Italy; danila.devito@uniba.it

**Keywords:** COVID-19, SARS-CoV-2, serological test, children, young adults, adolescents, family cluster

## Abstract

At the end of 2019, a new disease caused by the novel coronavirus SARS-CoV-2 appeared in Wuhan Province in China. Children seemed to be infected less frequently than adults, and family clusters seemed to play an important role in the spread of the pandemic. The aim of this study is to evaluate the serological profile of children and young adults between 4 and 16 years of age in order to assess the transmission patterns of COVID-19 between cohabitants. The subjects lived with at least one cohabitant who tested positive for the disease using a nasopharyngeal swab. To avoid contact with the disease, families were interviewed by telephone. Forty-nine children and adolescents with a mean age of 11 years were then subjected to a rapid lateral flow chromatographic test. Of them, seven (14.3%) were immunoglobulin G (IgG)-positive, and four (8.2%) were immunoglobulin M (IgM)-positive. In total, 16.3% of the tested sample had antibodies against SARS-CoV-2: this may confirm the lower vulnerability of children to COVID-19, despite the small sample size. The time from the negativization of the cohabitant until the test day may have influenced the results, especially when this timeframe is wide.

## 1. Introduction

In the initial phase of the COVID-19 pandemic, Italy was one of the most affected countries in the world. Since the initiation of the outbreak on March 25, Italy has registered the second-highest number of infections [1].

The severe acute respiratory syndrome coronavirus 2 (SARS-CoV-2) is a beta-coronavirus that uses the angiotensin-converting enzyme II (ACE2) to infect host cells [2,3,4].

More specifically, viral entry is facilitated by the binding of the S1 unit of the viral spike protein (S) and the transmembrane protease serine 2 (TMPRSS2), expressed in lung alveolar epithelial cells, or the proprotein convertase Furin, which is also found in oral epithelial cells. This demonstrates the possibility to become infected through the lungs or oral cavity [5].

Once in host cells, the virus is able to alter the human immune response and influence white blood cells and lymphocytes [6,7].

Children appear to be less vulnerable to coronavirus, and if they do become infected, they often have milder symptoms or experience an asymptomatic state [8,9,10,11].

Previous hypotheses have tried to explain this decreased response to SARS-CoV-2. It could be due to less exposure to the external environment or to host factors, such as the different affinity and expression of their ACE2 receptors or their developing immune system [12]. For example, children seem to have reduced concentrations of proinflammatory cytokines and C-reactive protein (CRP), which could explain the lower levels of immune-mediated damage in children and their mild symptoms; moreover, the immune systems of children may be more resistant to some viruses due to their frequent exposure to respiratory infections [6,13,14,15,16,17].

Several studies have reported percentages of children affected by SARS-CoV-2 infection, ranging from about 1% in Italy [1,18], 2% in China [13], and 5% in the USA [19], to less than 5% worldwide in a more recent article [20].

When evaluating the spread of the virus in children, screening strategies should include both gastrointestinal and respiratory symptoms; otherwise, 40–50% of cases may be excluded [20].

As far as gender differences are concerned, studies indicate a higher prevalence of SARS-CoV-2 in males, but this difference is often not statistically significant [21,22]. Infection has been demonstrated in all ages, and infants appear to be more vulnerable [14,21].

In the context of person-to-person transmission, particular attention must be paid to family clusters [14,23], especially in generating data to support decisions about school attendance. The rates of exposure within family clusters were different in the various studies [1,22]. Cases of infected children in families with at least one infected member have frequently been reported [14,24,25,26,27]. More specifically, one study found that elderly relatives were more likely to become infected first and then spread the infection to the other family members [17]. Another recent American study analyzing family clusters found that children and adults (with a positive cohabitant) had similar rates of infection, but children developed fewer and mostly nonspecific symptoms and milder illness [11].

Children could therefore be infected by their relatives and must be considered, even if asymptomatic, as potential sources of contagion, thus playing a fundamental role in the spread of the infection [10,14].

As far as the severity of clinical illness, children show a milder disease or they are asymptomatic in more than 90% of cases [6,14,15,20,21,28]. However, it is worth noting that severe manifestations have also been observed, particularly in children with comorbidities [10,14,17,22,29,30].

A systematic review published in June 2020 reported that 3% of cases showed severe symptoms (such as dyspnea, cyanosis of central origin, and hypoxia) and 1% of cases had critical conditions (i.e., respiratory failure or crisis, shock, and signs of multi-organ deficiency) [20].

Moreover, infants seem to have a higher risk for more severe symptoms [10,14,21,22]. Similarly, children with coexisting conditions, such as diabetes or asthma, or with a weak immune system, may develop more severe symptoms of the disease [1,10,14,22].

In general, the common symptoms of COVID-19 are less frequent in children (40–60%) [20,22], and can include fever, headache, mild cough, sputum (more common than in adults), runny nose, upper respiratory tract infections, gastrointestinal symptoms (diarrhea, nausea, vomiting), tachypnoea, tachycardia, and pharyngeal erythema [10,11,17,20,28,31]. Gastrointestinal symptoms are common, and they can be the only manifestation of COVID-19 in children; otherwise, they may arise after respiratory symptoms (as well as in adults) [20]. It also appears that the virus takes longer to clear the digestive tract [17].

Other symptoms such as poor appetite, abdominal pain, fatigue, myalgia, increased sweating, and dizziness were rarely observed [10,17,28,31]. Further studies are needed to evaluate the presence of anosmia and dysgeusia in pediatric patients [4].

Regarding the incubation period, it appears to be longer in children (6.5–7.5 days) than in adults (5.4 days) [4].

Finally, mortality is lower (<0.1%) than that of adults (5–15%) [20].

As far as computed tomography (CT) findings, even children may show positive CT images. Patchy infiltrates and consolidation, ground-glass opacities, or interstitial abnormalities may be observed, especially in symptomatic patients [4,17]. However, they seem less frequent than in adults [17].

According to Sun et al. [17], there are four possibilities:

- Symptoms and positive CT images (54%)
- Symptoms and absence of CT images (23%)
- Absence of symptoms and positive CT images (6.7%)
- Absence of both symptoms and CT images (23%).

Finally, since children of all ages are vulnerable to COVID-19, it is worth noting that, especially if asymptomatic, they can be considered carriers of the virus, thus contributing to its spread. Early identification of these children is therefore essential, and screening tests are increasingly important [17].

The aim of this study is to evaluate, through a qualitative detection of IgG and IgM antibodies to SARS-CoV-2, the impact of COVID-19 on people between 4 and 16 years old who belong to families with at least one positive swab result for the virus. Evaluation of the serological profile of this sample could allow us to assess the rate of contagion of children directly exposed to a known positive family member within a family cluster.

## 2. Materials and Methods

### 2.1. Study Design and Population

This was a population-based observational study. The protocol of the current study was registered on ISRCTN and is available with the following registration number: ISRCTN91064601.

The analyzed sample included children between 4 and 16 years of age who belonged to families with at least one positive swab result for COVID-19 and living in one of the five municipalities of the Milan Metropolitan Area: the districts of Segrate (MI), Vimodrone (MI), Peschiera Borromeo (MI), Crema (CR), and Lodi (LO). Through a collaboration between the University of Milan and these districts, the Azienda Socio-Sanitaria Territoriale (ASST) provided a list of positive subjects with cohabiting children, attributing to each family, adult, and child a code to prevent disclosure of their names in order to protect their privacy. Legal representatives of the children provided informed consent after the research aims and procedures had been explained to them. Participation in the study was voluntary.

**Figure 1:**
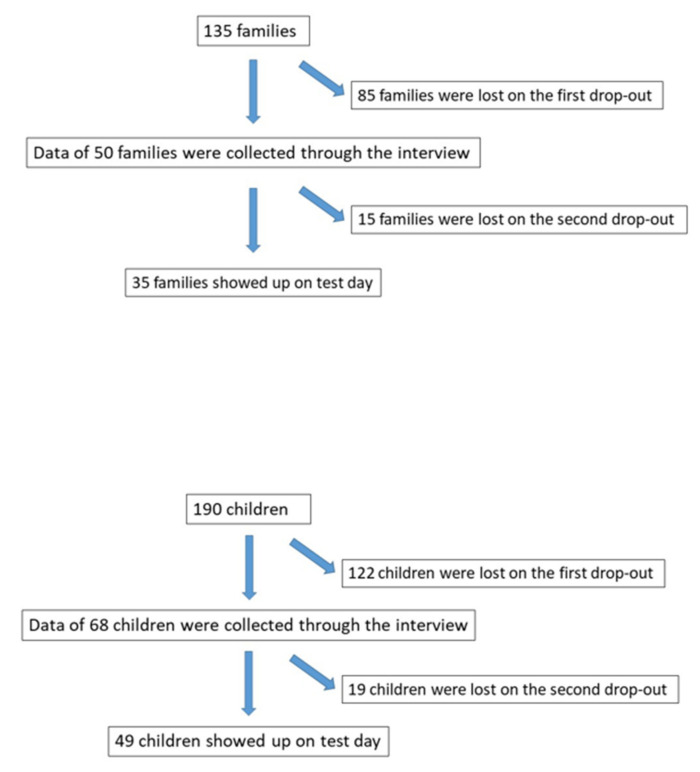
Drop-out flow chart.

We originally called 18 families (25 children) from Segrate, 3 families (6 children) from Vimodrone, 5 families (5 children) from Peschiera Borromeo, 44 families (54 children) from Crema, and 65 families (100 children) from Lodi, for a total of 135 families. However, some of them were unreachable by phone, some refused to participate, and some answered the interview but did not show up on test day. The reasons for drop-out are summarized in Figure 1.

Only subjects between 4 and 16 years old with at least one cohabitant who had been positive for SARS-CoV-2 according to a real-time Reverse Transcription-Polymerase Chain Reaction (real time RT-PCR) were considered for inclusion.

Subjects were excluded if they were younger than 4 or older than 16, if their household lacked at least one cohabitant with a previous positive swab result, or if their legal representatives did not provide informed consent.

### 2.2. Clinical Evaluation

Each family was interviewed by telephone. The interview was divided into three sections with a total of 24 questions: 9 questions about the family cluster, 8 questions about the subject who tested positive, and 7 questions about children between 4 and 16 years old. More specifically, the interview included questions about age, sex, number of cohabitants, symptoms, risk exposure during lockdown, swab results of other cohabitants (when performed), the course of the disease in the positive subject, drug therapy, and any chronic disease in the child. The interview is available as Supplementary Materials.

After the interview, families were contacted to arrange a rapid serological test of the child. Tests were carried out by health-care professionals from June 2020 to August 2020 on the following dates: June 13 (Segrate), June 25 (Vimodrone), July 28 (Peschiera Borromeo), July 30 (Crema), and August 6 (Lodi).

For this purpose, we used the Livzon Diagnostic Kit for IgM/IgG antibodies to Coronavirus (SARS-CoV-2). This is a rapid lateral flow chromatographic test which was first used in China. It qualitatively detects IgM and IgG antibodies to SARS-CoV-2 in human whole blood, plasma, or serum in vitro. It includes IgM and IgG test cassettes. If the test sample contains IgM or IgG antibodies to SARS-CoV-2, the test displays two different visible bands (test line and control line); however, if these antibodies are absent, only the control line appears.

The test consists of four steps: pricking the subject’s finger and collecting the blood, inserting one drop of the collected blood into sample wells, adding two drops of buffer, and reading the results. The wait time for the interpretation of the results is approximately 15 min.

### 2.3. Statistical Analysis

T test and Mann Whitney test were used to evaluate the differences between tested and untested groups in quantitative variables, while Chi-squared test and Fisher’s exact test were used to evaluate the differences in categorical variables.

Number, percentage, and the related exact confidence interval at 95% (CI95%) of IgG and IgM positive children were reported.

Considering that some children came from the same family, univariable logistic regressions with clustered standard errors were performed to evaluate possible factors associated with positive IgG. Odd Ratio (OR) and the CI95% have been reported.

Stata 16.1 (StataCorp LLC., TX, USA) [32] was used for all the analysis. A P-value less than 0.05 was considered statistically significant.

## 3. Results

Fifty families with a child or adolescent between 4 and 16 years old and at least one cohabitant with a previous positive SARS-CoV-2 swab result (for a total of 52 positive subjects) were interviewed. However, 15 families did not report on test day.

The mean age of positive adults was 47 years old (10.4 sd), with age ranging from 20 to 92 years old. Males made up 51.9%, as shown in Table 1.

At the time of investigation, the median value (Q1–Q3) of the time elapsed from diagnosis was 123 days (82–133) (this value is an approximation, since interviewed subjects did not always accurately remember the day they were diagnosed). Six subjects reported that they were still experiencing symptoms, although five of those had already received negative swab results between April 16th and May 22nd and were declared cured.

The number of cohabitants varied between two and seven, with a mean value (sd) of four (0.96). Of the interviewed families, 60% included symptomatic individuals who were not swabbed, shown in Table 2.

There were 68 children between 4 and 16 years old, with a mean age (sd) of 11 years old (3.5); 44.1% of them were male (Table 3). As Table 3 shows, only 7.4% came into contact with non-cohabitants during lockdown; 32.4% had taken medication in the last five months, and, at the time of the interview, just one child reported a runny nose, which is not one of the most common symptoms of COVID-19.

Serological tests were performed on 49 of the 68 children (79%). They were carried out between 22 and 152 days (median 98, Q1–Q3 80–135) after the adult’s diagnosis and between 7 and 134 days after the adult’s negativization (median 73, Q1–Q3 46–113).

Test and surveys were performed between June and August. More specifically, 28 serological tests (57%) were conducted in June and 21 (43%) between the end of July and the beginning of August. Each subject was tested once.

Of the 49 children tested, 7 (14.3% CI95% 5.9%–27.2%) were IgG-positive, while 4 (8.2% CI95% 2.3%–19.6%) were IgM-positive. A total of eight children were found to be positive using the serological test (16.3%): five females and three males.

The logistic regression on IgG positivity did not show a statistically significant influence by the evaluated variables. However, subjects tested more than 73 days after the adult negativization showed a lower probability of receiving a positive result (*p* = 0.059), as shown in Table 4. This finding is almost significant.

In June, 57% of tests were carried out, while the other 43% were performed at the end of July and in early August. Considering that the serological tests carried out in June had a maximum of 87 days from negativization, we focused on this subgroup. The time from the negativization of the positive cohabitants in tests carried out in June ranged between 7 and 87 days (median 50. Q1–Q3 42–62), and the time from the diagnosis ranged between 22 and 105 days (median 86, Q1–Q3 71–97).

In June, 28 serological tests (out of 29 interviewed children) were performed on subjects from 18 families. As Table 5 shows, six children (21.4%, CI95% 8.3%–41.0%) were IgG-positive and four (14.3% CI95% 4.0%–32.7%) were IgM-positive. A total of seven children returned positive results in the serological test (25%): four females and three males. No statistically significant relationship between the evaluated variables and IgG positivity was found in this subsample.

Three out of five children of at least 14 years of age tested positive, making up 50% of the total positive swabs. 

This could indicate that younger children may be less vulnerable to SARS-CoV-2 infection; however, the small sample size does not allow us to consider this result as being statistically significant.

The OR of ≥14 years old vs. <14 years old that was obtained from the logistic regression on IgG positivity is 10 (CI95% 1.03–97.07 *p* = 0.047).

## 4. Discussion

From the initial 135 families identified by ASST, there were two drop-out events in this study. The first included families who did not participate in the telephone interview, totaling 85 families, or 122 children, who were excluded from the research study. The second drop-out event included families that participated in the telephone interview but did not report on test day; this was the case for 15 families and 19 children. The tested group therefore included 35 families and a total of 49 children out of 68 (79%). The high drop-out level was mainly due to fear among the subjects of the quarantine measures and potential consequences of communicating positive results to health authorities.

Rapid lateral flow chromatographic tests were performed, which aimed to qualitatively detect IgM and IgG antibodies to SARS-CoV-2. The current gold standard for diagnostic detection of SARS-CoV-2 virus is the virus nucleic acid real time RT-PCR test. Yet, it requires expensive equipment and laboratories and several hours of processing time. To screen patients in the field, especially if they are asymptomatic, serological tests represent a valid alternative, being sensitive, specific, rapid, and simple [33]. They use the immune response to detect whether and approximately when a subject has been exposed to a specific pathogen. IgM is the first antibody to appear after exposure to an antigen. Its blood values remain high until IgG, which is more specific, is produced [33,34]. There are several serological assays now available to detect these antibodies: enzyme-linked immunosorbent assay (ELISA), neutralization assay, chemiluminescent immunoassay, and rapid diagnostic tests (RDT).

Among these, the rapid immunoglobulin M (IgM)—Immunoglobulin G (IgG) combined antibody test (RDT) is a lateral flow assay used to qualitatively detect IgG and IgM antibodies to SARS-CoV-2 from blood samples in a very limited amount of time. It requires approximately 10 to 30 min in a colloidal gold-based immunochromatographic strip assay [33,34,35]. Li et al. [33], among the first to develop a rapid kit specific to the new COVID-19 infection, demonstrated its usefulness in community surveillance, because it requires minimal training and is rapid while maintaining high specificity and sensitivity (respectively 90.63% and 88.66%). Moreover, compared to swabs, the possibility of detection of antibodies can highlight the progression of COVID-19 disease, and, since it consists of a blood collection, it reduces the risk of aerosol exposure to technicians [34].

The analysis of the results revealed a limited number of children and young adults with antibodies against SARS-CoV-2 (16.3%), despite each of them having a positive cohabitant. This could confirm the lower vulnerability of younger subjects to SARS-CoV-2 infection [8,9,10,11]. These results are consistent with a large, nationwide, population-based Spanish study, which involved more than 61,000 participants and found a lower seroprevalence in children and young adults than adults (3.4% of subjects between 0 and 19 years old compared to 4.4–6.0% of adults) [36].

A parameter that could influence the results is the time elapsed from the negativization of the positive cohabitant until the day of the serological test, especially if it is too long (the maximum value reached was 134 days). The results relating to the first period of the research (tests carried out in June), in which the maximum value was 87 days, showed that, in this case, the time elapsed from the negativization until the test was irrelevant. As stated by Kweon et al. [37], IgG antibodies remain at high levels even after 22–35 days from the onset of symptoms. However, our results indicated that, after a prolonged period of time, even IgG antibodies tended to be undetectable, and subjects showed negative results to the serological test. This seems to be true in cases starting from low antibody titers, as demonstrated in the study by Wajnberg et al. [38]. This, along with the small sample size and the lack of comparison with the other adult cohabitants of the same family cluster, may justify the difficulty in drawing firm conclusions and having significant results.

The literature shows that children often develop a milder disease or an asymptomatic state [6,14,15,20,21,28]. Most children who tested positive had no symptoms at the time of the interview. However, this does not exclude the fact that they may have previously developed the disease with related symptoms, so we cannot state with certainty that they were asymptomatic subjects.

Even children with coexisting conditions, such as asthma, which, according to the literature, may lead to a more severe disease [1,10,14,22], reported no symptoms at the time of the interview.

Finally, this study did not show significant differences between males and females, confirming some results of the previous literature [21]. In contrast, age may play a more important role: 50% of positive subjects were at least 14 years old. This could suggest lower vulnerability of toddlers to SARS-CoV-2 infection; however, the sample size was too small to consider this result statistically significant. Other limitations of this study are the absence of detailed information about symptoms of the children starting from the date of diagnosis of the positive cohabitant, as well as the use of a diagnostic kit which qualitatively evaluated the serological profile. For this reason, it is less reliable than serological tests that qualitatively and quantitatively detect antibodies. On the other hand, we believe it was appropriate for our children-based sample, due to the lower compliance of the subjects. Moreover, this was one of the first Italian studies focusing on children and adolescents within their family clusters, allowing us to analyze high-risk subjects living with people who have tested positive for SARS-CoV-2 using a nasopharingeal swab.

## 5. Conclusions

Only 16.3% of the sample showed the presence of antibodies against SARS-CoV-2. The days between the negativization of the positive cohabitant and the serological test of the child may have influenced the results: this may suggest that the antibody titer could become undetectable over time.

Most children who tested positive for coronavirus antibodies were asymptomatic at the time of interview, even if they had coexisting conditions such as asthma.

There were no significant differences between males and females, while younger children appeared to be less vulnerable to infection. We consider these results as preliminary due to the small sample size, pending future analysis that will allow us to draw firm conclusions.

## Figures and Tables

**Table 1:** Characteristics of positive cohabitants.

N		52	
Age (mean, sd)		47	10.4
Gender (*n*,%)			
	Female	25	48.1
	Male	27	51.9
Days from diagnosis ^ (median, Q1–Q3)		123	82–133
Days between symptoms and diagnosis (*n* = 49) (median, Q1–Q3)		8	4–17
Duration of disease * (*n* = 51) (median, Q1–Q3)		36	28–50
Hospitalization (*n*,%) (*n* = 47)		26	55.3
Symptoms at the time of the interview (*n*,%)		6	11.5
Asymptomatic (*n*,%)		4	7.7

^ at the time of the interview; * between the onset of symptoms (or diagnosis when the previous data were not reported) and negativization.

**Table 2:** Characteristics of families.

N		50	
Number of cohabitants (mean,sd)		4	0.96
Distribution number of cohabitants (*n*,%)			
	2	2	4.0
	3	10	20.0
	4	28	56.0
	5	6	12.0
	6	3	6.0
	7	1	2.0
Cohabitants 4–16 years old (*n*,%)			
	1	35	70.0
	2	13	26.0
	3	1	2.0
	4	1	2.0
Cohabitants <4 years old		7	14.0
Other cohabitants with positive swab		2	4.0
Symptomatic cohabitants who did not receive a swab		30	60.0

**Table 3:** Characteristics of children.

N		68	
Age (mean,sd)		11	3.5
Gender (*n*,%)			
	Female	38	55.9
	Male	30	44.1
Contact with non-cohabitants during lockdown		5	7.4
Chronic disease		3	4.4
Medication in the last five months		22	32.4

**Table 4:** IgG positivity in children tested between June and August (univariable logistic regression with clustered standard errors).

		OR	CI 95%	*p*
**Children Data**				
Age		1.12	0.86–1.45	0.397
Gender				
	Female	1		
	Male	1.22	0.26–5.70	0.802
Contact with non-cohabitants during lockdown				
	No	1		
	Yes	2.17	0.36–13.1	0.400
Chronic disease				
	No	1		
	Yes	6.83	0.38–122.0	0.191
Medication in the last five months				
	No	1		
	yes	1.28	0.22–7.43	0.783
Period				
	June	1		
	July/August	0.18	0.02–1.49	0.113
Days between diagnosis ^ and serological test		0.99	0.97–1.01	0.263
Days between negativization ^ and serological test		0.98	0.96–1.00	0.064
Days between negativization and serological test				
	≤73	1		
	>73	0.13	0.02–1.08	0.059
**Families Data**				
# cohabitants		1.12	0.73–1.73	0.600
# cohabitants 4–16 years old		1.41	0.87–2.28	0.164
Cohabitants with symptoms who did not receive a swab				
	No	1		
	Yes	1.87	0.42–8.44	0.413
**Positive Adult Data**				
Age		1.06	0.96–1.17	0.265
Gender				
	Female	1		
	Male	2.29	0.45–11.62	0.320
Days between symptoms and diagnosis (*n* = 49)		1	0.97–1.04	0.921
Duration of disease * (*n* = 51)		1.02	0.99–1.05	0.171
Hospitalization				
	No	1		
	Yes	5.4	0.67–43.58	0.113
Symptoms at the time of the interview (*n*,%)				
	No	1		
	Yes	1.7	0.35–8.20	0.509

^ of the positive adult; * between the onset of symptoms (or diagnosis if the subject did not tell us an onset date for symptoms) and negativization.

**Table 5:** IgG positivity in children tested in June (univariable regression with clustered standard errors).

		OR	CI 95%	*p*
**Children Data**				
Age		1.24	0.83–1.86	0.292
Gender				
	Female	1		
	Male	3.4	0.63–18.29	0.154
Contact with non-cohabitants during lockdown				
	No	1		
	Yes	2	0.37–10.68	0.417
Chronic disease				
	No	1		
	Yes	4.2	0.23–77.23	0.334
Medication in the last five months				
	No	1		
	Yes	1.33	0.20–8.94	0.767
Days between diagnosis ^ and serological test		1.01	0.97–1.06	0.509
Days between negativization ^ and serological test		1	0.96–1.04	0.903
Families data				
# cohabitants		0.97	0.58–1.61	0.894
# cohabitants 4–16 years old		1	0.56–1.79	1.000
Cohabitants with symptoms who did not receive a swab				
	No	1		
	Yes	3.46	0.58–20.74	0.174
Positive adults data				
Age		1.08	0.94–1.24	0.258
Gender				
	Female	1		
	Male	1.14	0.20–6.52	0.881
Days between symptoms and diagnosis		0.98	0.94–1.02	0.395
Duration of disease *		1.01	0.98–1.04	0.655
Hospitalization				
	No	1		
	Yes	3.46	0.40–29.6	0.257
Symptoms at the time of the interview (*n*,%)				
	No	1		
	yes	1.6	0.33–7.68	0.557

^ of the positive adult; * between the onset of symptoms (or diagnosis if the subject did not tell us an onset date for symptoms) and negativization.

## Data Availability

The data presented in this study are available on request from the corresponding author. The data are not publicly available due to the need to obtain the consent from mayors, who are the local health authorities.

## Supplementary Materials

The following are available online at https://www.mdpi.com/1660-4601/18/4/1488/s1, document S1: interview.
