# Supplementary material for: Serological Profile of Children and Young Adults with at Least One SARS-CoV-2 Positive Cohabitant: An Observational Study
Source: Int J Environ Res Public Health. 2021 Feb 4;18(4):1488. doi: 10.3390/ijerph18041488 (PMC7914765; doi:10.3390/ijerph18041488)
Supplement: Supplementary file 1 [file ijerph-18-01488-s001.pdf]

The interviewed subject was the positive cohabitant

Questions about the family cluster:

1) How many cohabitants are there in your family?
2) How old is the child’s father?
3) How old is the child’s mother?
4) How many cohabiting people are between 4 and 16 years old and how old are them?
5) How many cohabiting people are under 4 years old and how old are them?
6) How many cohabiting people are over 16 years old (without considering parents) and how old are them?
7) How many other cohabitants (apart from you) had a positive COVID-19 swab result?
8) How many cohabitants had negative COVID-19 swab results?
9) How many symptomatic cohabitants who did not receive a swab are there?

Questions about cohabitants who tested positive:

1) Which is your gender?
2) How old are you?
3) On which day were you diagnosed with COVID-19 via positive swab?
4) On which day did you have the first symptoms?
5) Have you been hospitalized?
6) Have you had a negative swab result stating that you are cured?
7) What day was the negativization formalized?
8) Do you currently have symptoms?

Questions about children:

1) How old is the child / young adult?
2) Which is the child / young adult’s gender?
3) Was the child / young adult in contact with non-cohabiting people during the lockdown period (grandparents, babysitters, etc...)?
4) Does the child / young adult have chronic diseases?
5) If the previous answer was positive, does the child / young adult take any medication for the current diseases?
6) What medication has the child / young adult taken in the last 5 months?
7) Does the child / young adult currently have symptoms?
